# A New Lens on Glycolipid Isomers in Spatial Neurobiology

**DOI:** 10.1021/acscentsci.6c01334

**Published:** 2026-09-09

**Authors:** Peggi M. Angel

**Affiliations:** Department of Pharmacology and Immunology, Proteomics Center, 2345 Medical University of South Carolina, Charleston, South Carolina 29425, United States

## Abstract

Integrating phospholipase C tissue pretreatment, MALDI-2 post-ionization, and optimized trapped ion mobility spectrometry to resolve glycolipid isomers within intact brain tissue.

## Graphical abstract

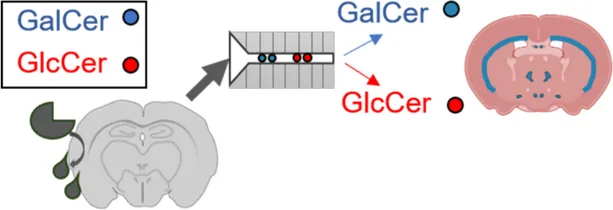

TThe extraordinary complexity of the brain extends far beyond its cellular architecture to a remarkable spatial diversity of molecules that defines neuronal function and forms the biological basis of human cognition and identity. Among these molecules, glycolipids occupy a unique position, serving not only as structural components of cell membranes but also as regulators of cell signaling, development, aging, and neurodegeneration.^1–3^ Innovation in spatial lipidomics by mass spectrometry has transformed our ability to investigate the brain and has led to the capability to visualize glycolipid distributions that underpin the molecular architecture of neuronal regulation.^4^ Yet, distinguishing glycolipid isomers directly in tissue while maintaining spatial information remains an analytical challenge. In a recent issue of *ACS Central Science*, Sweedler and colleagues^5^ provide an elegant solution by integrating enzymatic tissue preparation and trapped ion mobility spectrometry (TIMS) into a MALDI mass spectrometry imaging (MSI) workflow capable of resolving glycolipid isomers while preserving the intricate spatial organization of the brain (Figure 1). Their work represents an important step forward for neuronal spatial lipidomics and, more broadly, for the field of spatial glycoscience.

**Figure 1:**
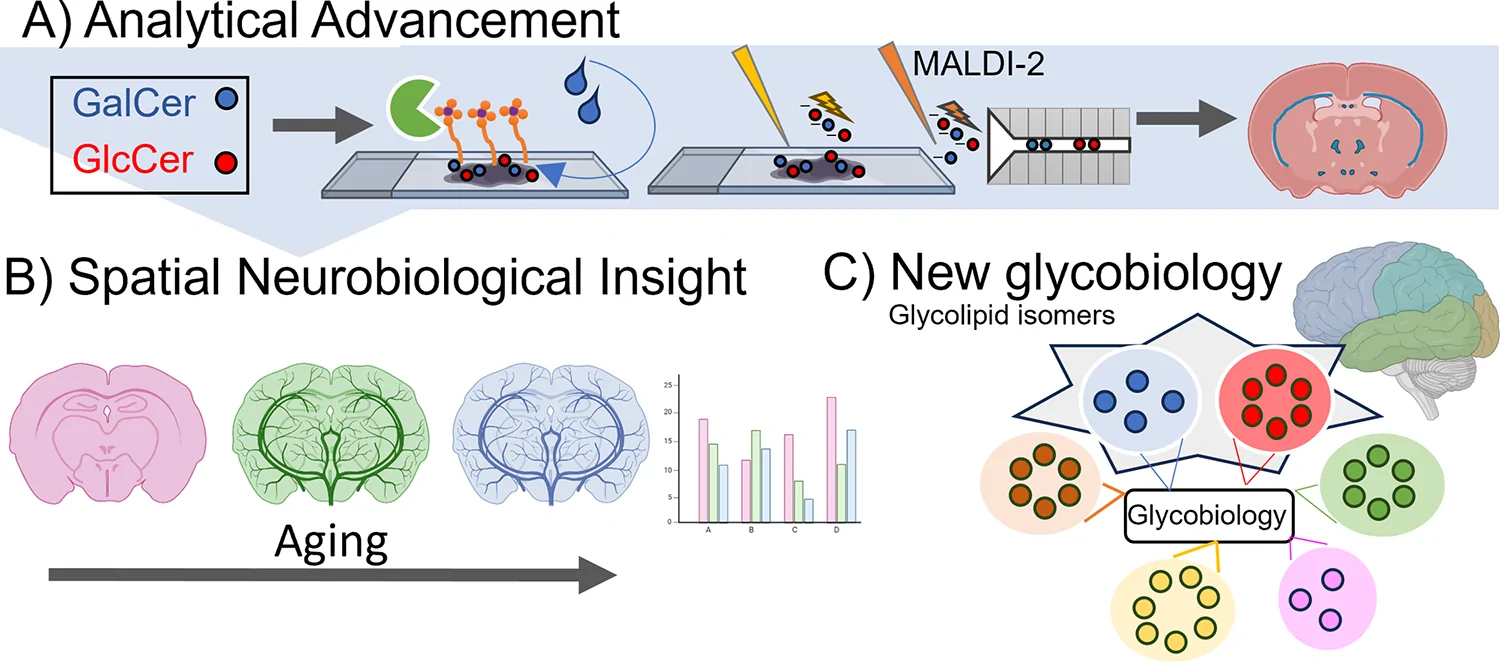
Analytical advancements lead to new biological insight. A) Analytical workflow development allowed detection of glycolipid isomers directly from tissue and maintaining spatial localization. B) The new approach was applied to brain aging, showing functionalized compartmentalization during aging. C) Glycolipid isomers add new information to the field of glycobiology.

Distinguishing glycolipid isomers directly in tissue while maintaining spatial information remains an analytical challenge. In a recent issue of *ACS Central Science*, Sweedler and colleague provide an elegant solution by integrating enzymatic tissue preparation and trapped ion mobility spectrometry (TIMS) into a MALDI mass spectrometry imaging (MSI) workflow capable of resolving glycolipid isomers while preserving the intricate spatial organization of the brain

Mass spectrometry imaging has transformed our understanding of tissue biology, allowing hundreds to thousands of molecular species to be visualized simultaneously without labels. However, imaging experiments are frequently constrained by a known limitation, that molecules with identical masses often cannot be distinguished from one another. For glycolipids, this limitation is particularly problematic because small structural differences can correspond to fundamentally different biological functions. Galactosylceramides (GalCer) and glucosylceramides (GlcCer), for example, differ only in the stereochemistry of a single monosaccharide, yet they occupy distinct metabolic pathways and play different physiological roles. Conventional MSI that maps only the precursor shows these molecules as a single signal and can mask deeper biologically meaningful information.

The analytical strategy developed by Chao et al.^5^ demonstrates that delving into structural variation can reveal an entirely different picture of glycolipid biology. Using phospholipase C (PLC) pretreatment to reduce ion suppression from phospholipids^6,7^ with careful tissue sample preparation, MALDI-2 postionization, and optimized TIMS separation, the authors successfully resolve GalCer and GlcCer directly from brain tissue sections. When imaging the complex tissue microenvironment, glycerophospholipids (GPLs) preferentially ionize and limit detection of other bioactive lipids. The use of PLC removes the ionizing phosphate headgroup from GPLs, resulting in an increased detection of the target glycolipids and represents how endogenous enzymes can be leveraged as analytical tools. Tissue preparation wash steps further increased the sensitivity and specificity by removing ionization suppressors including the PLC enzyme and native salt from the tissue. The contribution of MALDI-2 resulted in a more consistent detection of glycolipids following PLC treatment to improve analytical robustness. Importantly, both positive ionization mode and negative ionization mode were tested and it was the negative ion mode that resulted in improved mobility resolution 2-fold (fwhm) and achieved a resolving power for the isomeric glycolipids. Connecting these advances with the careful improvements to trapped ion mobility that optimized funnel-in pressure and ramp times to minimize fragmentation of the fragile GalCers and GlcCers resulted in a significant improvement in both detection sensitivity and specificity for glycolipids. The result was 47 glycolipid species detected with greater specificity in the brain across models of aging.

This study represents an excellent example of how innovation in methodology can fundamentally change the biological information addressed by mass spectrometry.

One of the most important aspects of this study is the focus on preservation of spatial information by the analytical workflow. Mass spectrometry imaging has transformed spatial analysis, allowing unlabeled complex molecular measurements to be interpreted within anatomical context rather than as averaged tissue homogenates. Here, resolving glycolipid isomers revealed distinct regional distributions of GalCer and GlcCer supporting functional segregation between the two isomers. Tracking the metabolic pathways driven by endogenous lipid enzymatic processing downstream of each precursor suggested a significant role for lipid enzyme activity within neuronal compartments. The application of the analytical workflow to understand neuronal aging showed that these spatially resolved metabolic pathways also change temporally. The authors report a selective decline in GalCer-associated metabolism while GlcCer remained comparatively stable across a rodent model of the aging brain. This distinction would have been obscured had the isomers been analyzed without a focus on isomeric separation. The work elegantly illustrates that resolving structural isomers can dramatically increase our understanding and interpretation of spatial biology.

From the perspective of glycobiology, the study also highlights an exciting advancement. Glycolipids represent an important cell interface along with glycosylation patterns in membrane biology, yet have often occupied a separate analytical space from glycoproteins and the study of glycans. As technologies advance, these boundaries are rapidly disappearing and are further being joined into multimodal analytical workflows that can identify how glycomolecules work together in tissue form and function.

The ability to spatially analyze sugar-containing molecules, including N-glycans, glycopeptides, glycoproteins, and now isomer-resolved glycolipids within the same mechanistic framework opens new opportunities.

This workflow would combine very well with metabolomic and transcriptomic spatial workflows^8^ to construct comprehensive maps of tissue glycobiology that distinguish disease states. Particularly for neuro, this would help understand biological networks directing the complex neuronal interface that underlies neuronal function. There is also translational opportunity in defining region-specific metabolic alterations before overt pathology develops. Spatial studies of isomeric glycolipids that additionally include proximal fluid analysis would provide momentum to develop new noninvasive biomarkers of compartmentalized disease emergence, progression, or therapeutic response. As such, further integration of glycobiology with glycolipid isomers deepens opportunities for precision medicine.

Analytical chemistry has always advanced by taking on challenges to measure difficult analytes in complex mixtures. In this study, Chao et al. demonstrate that even a small stereochemical difference in a single monosaccharide can be detected and evaluated for distinct biological information when viewed through an appropriate analytical lens. The isomer-resolved MSI workflow offers an optimized way of visualizing the details of glycolipid metabolism within intact tissues and establishes a foundation for future studies on glycolipids in brain aging and neurodegeneration. As spatial biology continues to expand, analytical approaches that bring together molecular specificity with anatomical context, such as the one presented here, have an increasingly central role in understanding the molecular organization of healthy and diseased tissues.
